# Marine-Derived Peptides with Anti-Hypertensive Properties: Prospects for Pharmaceuticals, Supplements, and Functional Food

**DOI:** 10.3390/md22040140

**Published:** 2024-03-22

**Authors:** Mari Johannessen Walquist, Karl-Erik Eilertsen, Edel Oddny Elvevoll, Ida-Johanne Jensen

**Affiliations:** 1 Faculty of Biosciences, Fisheries and Economics, The Norwegian College of Fishery Science, UiT The Arctic University of Norway, N-9037 Tromsø, Norway; karl-erik.eilertsen@uit.no (K.-E.E.); edel.elvevoll@uit.no (E.O.E.); 2 Department of Biotechnology and Food Science, Norwegian University of Science and Technology (NTNU), N-7491 Trondheim, Norway

**Keywords:** hypertension, antihypertensive, Mollusca, Porifera, Echinodermata, Cnidaria, Chordata, Tunicate, ACE-inhibition, bioactivity

## Abstract

Hypertension, a major health concern linked to heart disease and premature mortality, has prompted a search for alternative treatments due to side effects of existing medications. Sustainable harvesting of low-trophic marine organisms not only enhances food security but also provides a variety of bioactive molecules, including peptides. Despite comprising only a fraction of active natural compounds, peptides are ideal for drug development due to their size, stability, and resistance to degradation. Our review evaluates the anti-hypertensive properties of peptides and proteins derived from selected marine invertebrate phyla, examining the various methodologies used and their application in pharmaceuticals, supplements, and functional food. A considerable body of research exists on the anti-hypertensive effects of certain marine invertebrates, yet many species remain unexamined. The array of assessments methods, particularly for ACE inhibition, complicates the comparison of results. The dominance of in vitro and animal in vivo studies indicates a need for more clinical research in order to transition peptides into pharmaceuticals. Our findings lay the groundwork for further exploration of these promising marine invertebrates, emphasizing the need to balance scientific discovery and marine conservation for sustainable resource use.

## 1. Introduction

Hypertension, defined as persistent excessive systolic/diastolic blood pressure (SBP/DBP) over 130/80 mmHg [1], is a major public health concern and is considered the leading modifiable risk factor for cardiovascular disease (CVD) and premature death globally [2]. Despite the widespread use of anti-hypertensive medications, the prevalence of hypertension has increased in recent years, and it has been estimated that more than 30% of adults worldwide have hypertension [2]. As for CVD in general, the risk for hypertension increases with the number of risk factors present [3,4]. While gender, age, and heredity are non-modifiable factors, lifestyle-related factors, such as unhealthy diet, lack of physical activity, overweight and obesity, and alcohol consumption, are modifiable and thus preventable. Drugs available to reduce blood pressure (BP) are often associated with adverse side effects, such as cough, loss of taste, nausea, oedema, flush, headache, increased urination, rapid pulse, dizziness, high levels of blood potassium, skin rashes, hematological disorders, and gastrointestinal disorders [5]. Although not fatal, these side effects can be severe enough to affect adherence or discontinuation to medication [6]. It is estimated that poor adherence to anti-hypertensive medications and persistently elevated BP account for a considerable proportion of CVD events, including angina, myocardial infarction, chronic heart failure, kidney failure, transient cerebral ischemic attacks and strokes, premature mortality and disability, and increased hospitalization [7]. Therefore, much effort has been put into developing natural compounds with fewer adverse effects.

A change in dietary patterns may be an effective approach to maintain good health, and the consumption of fish and seafood has been shown to be associated with a reduced risk of CVD events [8]. The beneficial health effects of seafood consumption have traditionally been attributed to its long-chain polyunsaturated fatty acids. Still, seafood is increasingly appreciated as a rich source of high-quality proteins containing taurine and all essential amino acids [9]. Extensive research has been conducted on low-trophic marine organisms, and especially marine invertebrates, including phyla such as Mollusca, Porifera, Echinodermata, Cnidaria, and Chordata) to uncover bioactive compounds [10]. Mollusca is one of the most species-rich phyla, comprising a wide range of terrestrial and aquatic organisms. Mollusks generally have a soft body mostly enclosed within a hard protective shell. Of the more than 80,000 described species, roughly 44,000 are habituated in seas worldwide [11]. Abalones, escargots, mussels, clams and cephalopods form a significant percentage of “seafood” [12]. The phylum Porifera comprises nearly 9000 sponges [13], multicellular organisms with bodies full of pores and channels allowing water to circulate through them. Most sponges are marine species, and the adult sponges are sessile from tidal zones to 8800 m depths. Sea urchins, sea cucumbers, sea lilies, sea stars, and brittle stars constitute a large and entirely marine phylum, Echinodermata, with approximately 7000 living species [14]. Adult forms of echinoderms are found at every depth, from the tidal zones to the abyssal zones, playing an important role in benthic ecosystems on the ocean floor. The phylum Cnidaria comprises more than 11,000 species including sea anemones, corals, sea pens, jellyfish, box jellies, hydroids and Staurozoa. Cnidarians mostly have two basic body forms: swimming medusae and sessile polyps, and stinging cells containing toxins for prey capture and defense—cnidae. Tunicates belong to the subphylum (Urochordata) in the phylum Chordata [15]. The tunicates have an external layer, a tunic, rich in collagen and tunicin, that embeds the body. Tunicates include both sessile species (ascidians) and pelagic species (Thaliacea and Appendicularia) [15].

Due to the permanence of many invertebrates, they have evolved defense mechanisms to survive, mitigate competition and deter potential predators, thereby presenting an opportunity to discover bioactive compounds. It is noteworthy that invertebrates exhibit the highest relative abundance of bioactive compounds Approximately 75% of all bioactive compounds found in the years 1985–2012 originated from invertebrates [10]. Most of these compounds had anti-cancer activity, accounting for 56% of all bioactive compounds. In contrast only 1% of these bioactive compounds were associated with the prevention of heart and vascular disease [10]. Many marine invertebrates are filter feeders that may retain large amounts of microorganisms within their bodies. Indeed, microorganisms, such as bacteria, fungi, cyanobacteria, and unicellular algae, may account for a large portion of the fresh weight of marine sponges [16,17] and these microorganisms are responsible for producing a substantial part of the secondary metabolites identified in such invertebrates [18]. Of all the discovered bioactive compounds between 1985 and 2012, approximately 9% were peptides [10]. Peptides are small, typically comprised of 2–20 amino acids, and offer higher stability and more straightforward synthesis than more complex natural compounds. Hence, they have significant potential for applications in pharmaceuticals and functional foods. Food proteins typically contain specific peptide sequences that, when released by enzymatic treatment, bacterial fermentation or upon digestion, may be sources of biologically active peptides with anti-hypertensive effects [19,20]. The bioactivity of these peptides is linked to their distinct structural characteristics, such as the sequence and composition of amino acids, the balance of hydrophobic and hydrophilic properties along the amino acid chain, the nature of the residues at the N- and C-termini, as well as the overall charge distribution conferred by the amino acids within the peptides [19]. These factors collectively dictate the peptides’ functional interactions with biological targets. This bioactivity can still be modified when passing through the gastrointestinal tract (GI). However, peptides with shorter amino acid sequences have demonstrated enhanced resistance to in vitro GI digestion [21]. This resilience is further supported by attributes such as reduced hydrophobicity, branched-chain aliphatic N-terminal residues, increased proportion of histidine and proline, and a more positive net charge at physiological pH [21]. Thus, these peptides are more likely to be transported to the bloodstream or reach target organs in their bioactive form, as opposed to their less stable counterparts. Bioactive peptides from commonly consumed fish and fish by-products have been extensively studied [22]. Harvest of low-trophic marine organisms has been suggested as a strategy to contribute to food security [23], and it is therefore relevant to map putative bioactive peptides from these sources. The utilization of these novel sources is not limited to being a mere protein source, as it also presents the opportunity to extract or isolate bioactive peptides that can be incorporated as ingredients in functional foods, food supplements, or synthesized as drugs.

Anti-hypertensive peptides can lower blood pressure through various mechanisms. One common mode of action is the inhibition of the angiotensin-converting enzyme (ACE), which reduces the production of the vasoconstrictor angiotensin II (Ang II), leading to vasodilation and decreased blood pressure [24]. Additionally, these peptides may promote the release of the important vasodilator nitric oxide (NO) from the endothelium, further contributing to vasodilation. Some peptides can inhibit renin, an enzyme crucial to the renin–angiotensin–aldosterone system (RAAS), thereby reducing Ang II levels. Others may act as antagonists to Ang II receptors, particularly the angiotensin (AT) 1 receptor, preventing Ang II from increasing blood pressure [24]. Beyond these mechanisms, anti-hypertensive peptides can also exert diuretic and natriuretic effects, helping the body to excrete excess sodium and water, which in turn can lower blood volume and pressure. Some peptides may directly induce vasodilation by interacting with vascular smooth muscle cells, independent of RAAS and the endothelial pathways. Finally, these peptides can modulate the autonomic nervous system, influencing the balance between the sympathetic and parasympathetic systems and affecting heart rate and vascular constriction [24]. While the exact pathways and effectiveness of anti-hypertensive peptides vary, ongoing research continues to explore their potential as therapeutic agents for managing hypertension. There are various research methods for investigating potential anti-hypertensive peptides. These include biochemical assays, cell-based assays, in silico approaches, and animal and human interventions, and these methods are all represented by articles included in this review.

In this study, we extensively reviewed the literature from 2000 to 2023, focusing on the anti-hypertensive activity of peptides and proteins derived from the principal phyla of marine invertebrates. We mapped the methodology employed and explored the suitability of the peptides and proteins for pharmaceutical applications and their potential as supplements, or ingredients in functional food.

### 1.1. Biochemical Studies

Biochemical assays are commonly used to investigate the putative anti-hypertensive activity of hydrolysates and isolated peptides, as these are affordable, time-saving, and readily accessible. Commonly employed assays for determining anti-hypertensive potential include those measuring ACE or renin inhibiting activity. In our review, we concentrate on the ACE inhibitory activity assay, which exists in various forms and has been extensively reviewed by Ahmad et al. [25]. These ACE inhibitory activity assays utilize different substrates in their reactions and employ a range of methods to evaluate the outcome of the enzymatic processes or to separate the substrates from the products. The techniques used for measurement and separation include fluorometric and spectrophotometric methods, electrophoresis, radiochemistry, and high-performance liquid chromatography [25]. The peptide concentration required to inhibit 50% of the enzyme activity (IC_50_ value) is a metric commonly used to compare the biological activity of hydrolysates or peptides across different studies. However, there are several challenges to this approach. In 2013, Ben et al. conducted a study examining ACE inhibitory assays, in which they used synthetic di- and tripeptides, as well as captopril as an ACE inhibitor and losartan as a competitive antagonist of the receptor, to highlight the differences in results [26]. The study used two widely recognized approaches for measuring ACE inhibition: a modified version of Cushman and Cheung’s assay with *N*-a-hippuryl-L-histidyl-L-leucin (HHL) as a substrate and another method using N-[3-(2-furyl) acryloyl]-L-phenylalanyl-glycyl-glycine (FAPGG) as a substrate. This study also included angiotensin-I-substrate from rabbit lungs for comparison with the synthetic substrate, and it was reported that the obtained IC_50_ values were not comparable across different substrates and methods [26]. It is essential to consider all relevant parameters when comparing IC_50_ values of hydrolysates or peptides to define biological activity, and failure to do so may lead to the formation of biased conclusions. The scientific articles included in this review primarily utilized commercially available enzymes to hydrolyze raw materials or bacterial fermentation (Table 1). Both hydrolysates and smaller peptides were investigated for their potential anti-hypertensive effects. Captopril was commonly used as a positive control, or various hydrolysates and peptides were compared based on their ACE inhibitory activity (IC_50_ values or % ACE inhibition).

Food-grade enzymes are commonly used to hydrolyze sources intended for human consumption. When hydrolyzing Mediterranean mussel (*Mytilus galloprovincialis*, Lamarck 1819) meat, the enzyme subtilisin resulted in higher ACE inhibitory activity compared to corolase [27]. In another study, the combined use of food-grade enzymes, namely Alcalase^®^, flavourzyme, and corolase PP, resulted in a higher ACE inhibitory activity compared to individual enzyme hydrolysis of meat derived from blue mussel (*Mytilus edulis,* Linnaeus, 1758) non-saleable co-products [28]. The blue mussel industry generates substantial amounts of by-products beyond these non-saleable co-products. Hydrolysis of blue mussel byssus, a collagenous protein strand, with corolase PP displayed ACE inhibitory activity higher than that of unhydrolyzed protein [29]. The processing of bivalves for food generates substantial quantities of shell by-products. Notably, a hexapeptide isolated from a hydrolysate of Akoya pearl oyster (*Pinctada fucata,* A. Gould, 1850) shells, when treated with orientase 22 BF protease, demonstrated promising ACE inhibitory activity [30]. This activity surpassed that of hydrolysates derived from nucleicin, indicating the potential for developing high-value bioactive compounds from what would otherwise be waste material. ACE inhibitory activity was also found in hydrolysates derived from the muscle of Akoya pearl oyster. In silico screening methods were used to identify potential active peptides, which were subsequently tested for ACE inhibitory activity [31]. Chinese Venus (*Cyclina sinensis,* Gmelin, 1791) was hydrolyzed with trypsin, resulting in the identification of ACE inhibitory peptides with a molecular weight of less than 3 kDa [32]. Another study used ultracentrifugation to fractionate hydrolysates from the muscle and hemolymph of West African mid creeper (*Tympanotonus fuscatus radula,* Linnaeus, 1758) and Nigerian periwinkles (*Pachymelania aurita*, O. F. Müller, 1774) [33]. The ultrafiltrates from these mollusks exhibited ACE inhibitory activity, although, significantly lower than the control (Captopril). In another study, enzymatic digestion with a combination of neutral protease and trypsin of the mantle tissue of *Chlamys farreri* (Jones and Preston, 1904) yielded a hydrolysate with ACE inhibitory activity [34].

In recent times, there has been increased attention toward green technology and eco-friendly hydrolyzation methods as a sustainable alternative to conventional enzymatic hydrolysis [47]. In silico strategies, supported by bioinformatic tools, have the potential to be more targeted and sustainable compared to traditional experimental approaches. Despite their efficacy in prediction, in silico methods do not synthesize the peptides; therefore, it is important to pair them with novel eco-friendly synthesis techniques for peptide production. For instance, switching to enzymatic hydrolysis from chemical methods already provides milder conditions, higher specificity, and eliminates the need for organic solvent and toxic constituents in peptide production. Nevertheless, the use of expensive enzymes with lower yield and the scarcity of food-grade enzymes have led to a search for novel technologies. These include membranes with immobilized enzymes, high-pressure systems, ultrasound, ohmic heating, pulsed electric fields, microwave-assisted extractions, and subcritical water hydrolysis (SCWH) [48,49]. Such technologies can be employed in tandem with enzymatic hydrolysis or as a pre-treatment to unfold proteins, denature them, or disrupt weak protein interactions. This enhances hydrolysis by increasing the accessibility of the target sites, facilitating the release of small peptides. Although the initial implementation cost is currently high for many of these technologies, the potential for future scaling up and the ability to integrate adaptations within the same process might offset this drawback. The comb pen shell (*Atrina pectinata*, Linnaeus, 1767) is a widely consumed bivalve known for its high-value proteins. Two separate studies have conducted SCWH to extract ACE inhibitory peptides from the muscle tissue [35] and viscera [36] of this bivalve. These hydrolysates exhibited high ACE inhibition, similar to 0.1% Captopril. However, although SCWH is a flexible and controllable process, the protein cleavage prediction and specificity remain unclear [47]. Further research and experimentation with reference proteins are necessary to fully unlock the potential of SCWH as an alternative to conventional hydrolysis methods.

Only one study has been conducted on the marine sponge *Stylotella aurantium* (Kelly-Borges and Bergquist, 1988). In this study, pepsin hydrolysis yielded two dipeptides exerting ACE inhibitory activity. The authors confirmed their findings by synthesizing the dipeptides and demonstrating that the ACE inhibitory activity was associated with hydrogen bonding and Pi interaction between the peptides and the ACE complex [37].

The edible sea cucumber stonefish (*Actinopyga lecanora*, Jaeger, 1833) belongs to the phylum Echinodermata and has been investigated for its ACE inhibitory activity. Among various commercial enzymes used for hydrolysis, hydrolysates prepared using Alcalase^®^ and bromelain exhibited the highest ACE inhibitory activity, surpassing hydrolysates prepared with papain, flavourzyme, pepsin, and trypsin [38]. However, a separate study involving underutilized Indonesian sea cucumbers found that hydrolysates prepared using Alcalase^®^ have stronger ACE inhibitory activity compared to hydrolysates produced with bromelain or a combination of the two enzymes [39]. This highlights the significance of exploring the influence of different enzymes, either individually or in combination, on the various raw materials in order to yield peptides with the highest anti-hypertensive activity. Proteins derived from the sea cucumber *Apostichopus japonicus* (Selenka, 1867) were utilized in a study that employed an integrated process technique called enzymatic membrane reactor to produce desalinated peptides after hydrolysis with Alcalase^®^ [40]. Desalinated peptides with ACE inhibitory activity were subjected to in vitro simulated digestion, resulting in a small reduction in their ACE inhibitory activity. Li et al. conducted a study to enhance the productivity of hypertensive peptides by adding exogenous amino acids to protein hydrolysates from the sea cucumber *Acaudina molpadioides* (Semper, 1867) [41]. The peptide that exhibited the highest level of ACE inhibitory activity had enhanced hydrophobicity due to the incorporation of exogenous proline.

The box jellyfish, *Chiropsalmus quadrigatus* (Haeckel, 1880), has poisonous tentacles containing potential bioactive peptides. Hydrolysates from a venom extract of this particular box jellyfish were generated using pepsin and papain [42]. The ACE inhibitory activity was tested in vitro, while the toxicity of the peptides was tested in vivo in SD rats. After employing ultracentrifugation and MALDI-TOF/MS analysis, a novel decapeptide was identified as having the most potent ACE inhibitory activity. *Rhopilema esculentum* (Kishinouye, 1891), also known as the flame jellyfish, is a species that is commonly consumed in Southeastern Asia. In a study by Liu et al., jellyfish hydrolysates produced using compound proteinase AQ exhibited higher ACE inhibitory activity compared to hydrolysates produced with pepsin, papain, Alcalase^®^, Protamex, or compound proteinase AN [43]. The peptide that displayed the highest ACE inhibitory activity was characterized by the presence of tyrosine at the C-terminal, which likely contributed to increased hydrophobicity [43]. In another study, collagen was extracted from *R. esculentum* and hydrolyzed with seven proteases [44]. Of the resulting hydrolysates, Alcalase^®^ produced the one with the highest ACE inhibitory activity, which was further evaluated in a subsequent in vivo study [50]. Two studies on the tunicates *Styela plicata* (Lesueur, 1823) and *Styela clava* (Herdman, 1881) using nine distinct proteases for hydrolyzation have revealed that the Protamex hydrolysates contained peptides with the most potent ACE inhibitory activity in both studies [45,46]. The strategic application of food-grade enzymes in the production of anti-hypertensive hydrolysates can unlock their immense potential as key ingredients in functional food and offer a dual advantage to producers and consumers alike.

### 1.2. Cell Studies

Advancing from biochemical assays to more physiologically relevant approaches is essential for investigating anti-hypertensive effects in a more relevant setting, and in vitro cell models hold significant importance for hypothesis testing. These models provide the ability to control experimental variables and to study mechanisms and molecular interactions at higher levels. As reviewed in Medina-Leyte et al., the human umbilical vein endothelial cell (HUVEC) is a known in vitro cell model used to study various diseases, including cardiovascular diseases [51]. The endothelium serves as a barrier between blood and organs and plays a vital role in regulating BP, blood flow, and coagulation through vasoconstriction by secreting NO and endothelin [51]. The association between oxidative stress and hypertension is widely recognized, as highlighted in the review by Griendling et al., which emphasizes the involvement of complex processes related to reactive oxygen species [52]. Despite these complexities, the HUVECs model remains valuable for evaluating specific reactive oxygen species, such as hydrogen peroxide. Consequently, the HUVEC model holds relevance in investigating the protective effects of anti-hypertensive drugs on the endothelium. This review identified two studies that utilized cell models without subsequent in vivo analysis, and both studies had HUVECs as their chosen cell model (Table 2).

When blue mussel proteins were subjected to hydrolysis with pepsin followed by trypsin, the hydrolysates exhibited significantly stronger ACE inhibitory activity compared to hydrolysates produced using pepsin, trypsin, or trypsin followed by pepsin [53]. Through molecular docking affinity testing, it was revealed that the most potent of these anti-hypertensive peptides interact with the active site of ACE through hydrogen bonding, electrostatic force, and hydrophobic interactions. In HUVECs, these peptides also increased NO and decreased endothelin secretion, adding to the protective effect. In a separate study, peptides with previously known ACE inhibitory activity derived from *Volutharpa ampullacea perryi* (Jay, 1857), a deep sea snail native to the Yellow Sea of China, comprised 50% proteins in the edible parts [54]. Molecular docking analysis revealed that these peptides primarily interacted with the ACE complex through hydrogen bonding, and the inhibitory interaction was found to correlate with the number of hydrogen bonds formed. When tested in HUVECs, these peptides exhibited an increase in NO secretion and a reduction in endothelin secretion, in addition to a protective effect against hydrogen peroxide-induced cell injury [54].

### 1.3. In Silico

In silico experiments refer to computer-based experiments that utilize computational models or databases to achieve desired results (Table 2). According to Kamble et al., conventional methods for bioprospecting are not only costly and time-consuming but also require significant resources [58]. In contrast, exploring biological databases using bioinformatics tools reduces resource demand and potentially increases the success rate [58]. However, it is important to note that this approach relies on the ability to test the predicted bioactive peptides and cannot fully account for dynamic factors present in the conventional screening process for novel bioactive peptides. Skin from jumbo squid (*Dosidicus* gigas, d’Orbigny, 1835) is an underutilized by-product from an economically important fishery in the regions of the Pacific Ocean and from southern Chile to the southern United States. Shotgun silico hydrolysis was used to characterize the skin proteome and to predict a high number of different peptides with various potential bioactivity, including anti-hypertensive [55]. This study gave an overview of the proteomics of the skin of the jumbo squid. However, the bioactivity is predicted in silico and not tested in vitro. Another study, hydrolyzing the myosin heavy chain in Japanese flying squid (*Todarodes pacificus*, Steenstrup, 1880), used both in silico and in vitro methods to investigate the potential of bioactive peptides [56]. Firstly, they hydrolyzed in silico with different enzymes, due to the estimated toxicity, allergenicity, gastrointestinal stability, and bioavailability, and nine novel peptides with short amino acid sequences were predicted. Secondly, these peptides were synthesized, but only two novel peptides exhibited strong ACE-inhibitory activity in vitro. This demonstrates the importance of testing the peptides in vitro to verify the predicted activity. Pacific oyster (*Crassostrea gigas*, Thunberg, 1793) is a widely cultivated and consumed mollusk, originally native to the western Pacific Ocean. Large proteins from the mollusk were subjected to in silico simulated gastrointestinal digestion to identify promising bioactive peptides [57]. To optimize the targeted search, the authors used databases to exclude peptides with potential toxic or allergenic effects, bitterness, or low bioavailability, resulting in five potential anti-hypertensive peptides that should be validated in vitro and in vivo.

### 1.4. Animal Studies

Several animal studies have been published on this subject (Table 3). The most widely used model for evaluating hypertension and anti-hypertensive effects involves spontaneously hypertensive rats (SHRs). These rats are bred specifically to develop high BP, making them well-suited for both acute and chronic studies. In this review, we observe that most articles examined the impact of marine proteins or peptides on BP, focusing on acute or chronic effects, while three studies investigated both the acute and the chronic effects in the same study. Peptides or marine hydrolysates are orally administered to SHRs using oral injection, gavage or as a feed ingredient, and BP measurements are conducted before administration, after, and at subsequent intervals throughout the studies. The documentation of anti-hypertensive effects from lower trophic species in SHRs dates to the mid-2000s. However, studies exploring the impact of cnidarian toxins on BP in different animals were published as early as the late 1960s [59,60,61].

Extensive research has been conducted on mussels, examining them in both fermented and hydrolyzed forms. Blue mussel meat was subjected to a 6 month fermentation process and a decapeptide that exhibited a notable increase in ACE inhibitor activity was identified and isolated from the sauce [62]. This decapeptide significantly reduced BP in SHRs after oral administration compared to control rats receiving a saline control solution. It is, however, worth nothing that the observed decrease, while significant, was not as effective as Captopril. In a separate study, SHRs were fed with hydrolyzed blue mussel meat (10 or 20 mg/kg/day) for 28 days. This intervention resulted in a reduction in BP accompanied by changes in the expression of genes for cytokines, growth factors, and genes involved in the blood coagulation system and fatty acid metabolism [63]. However, there were no significant alterations observed for genes involved in RAAS, a key player in BP regulation. Another relevant study involved administration of *Lactiplantibacillus pentosus* SN001 fermented Australian Blacklip abalone (*Haliotis rubra*, Leach 1814) viscera to 14-weeks old SHRs, constituting 5% of the diet for 9 weeks [64]. This intervention demonstrated a significant reduction in SBP compared to control groups receiving unfermented viscera or standard diet. An ACE inhibitory peptide from *Ruditapes philippinarum* (Adams and Reeve, 1850) fermented with *Bacillus natto* was found to enhance NO release, reduce EF-1 and ROS in HUVECs, and lower BP in L-*N*-nitroarginine pre-treated Sprague Dawley (SD) rats [65]. In a following study, the fermented product containing this peptide and more specified peptides significantly decreased SBP and DBP, and restored hypertension-related kidney damage, in SHRs after an 8 week administration at 100 mg/kg bw [66]. In a related endeavor, *Bacillus natto* was employed to ferment *C. farreri* mantle (skirt of scallop), leading to a hydrolysate containing five identified ACE inhibitory peptides. This resulting hydrolysate, evaluated using SHRs through single-dose and continuous administration, exhibited a significant reduction in SBP comparable to captopril in both experimental setups [67]. An aloase and pancitase hydrolysate from the meat of an undisclosed oyster, was fed to SHRs over a four week period revealing significant differences from the control after two and four weeks of treatment [68]. In another study, a trypsin hydrolysate from the oyster (*C. gigas*) led to a significant reduction in SBP after long-term feeding of the hydrolysate to SHRs [69]. In addition, a penta-peptide was isolated from the hydrolysate that significantly reduced SBP after a single administration to SHRs (50 mg/kg). Simulated in vitro digestion using pepsin and pancreatin of this pentapeptide also revealed a dipeptide with retained anti-hypertensive activity [69]. Transglutaminase cross-linked oyster protein (undisclosed species) was subjected to a two-step hydrolysis, yielding a hydrolysate containing five ACE inhibitory peptides [70]. This hydrolysate significantly reduced SBP in SHRs when given as a single bolus (100 mg/kg bw) compared to a commercially available sardine hydrolysate [70]. However, the study’s validity is limited by a small sample size of only three rats per experimental group. Hydrolysate from the meat of pearl oyster (*Pinctada fucata martensii*, Dunker 1880) contained two novel peptides and has been shown to reduce SBP in SD rats after a single bolus intravenous injection (10 mg/kg bw) [71]. This BP-reducing effect was comparable to captopril, although less potent; however, the duration of the rat study was only 45 min, and the authors did not disclose the sample size.

Enzymatic consecutive hydrolyses of body wall protein from the sea cucumber *A. molpadioides* using bromelain and Alcalase^®^ produced a novel decapeptide with ACE inhibitory activity [72]. The peptide retained its ACE inhibitory activity after in vitro gastrointestinal digestion, and when a single dose was orally administrated to SHRs (3 mM/kg), it significantly reduced SBP compared to the control group. SD rats were pre-fed a hydrolysate in various doses from the sea cucumber stonefish (*A. lecanora*), in rats already induced with hypertension via angiotensin I injection [73]. The integrity of this study may be questioned, as the authors failed to disclose the sample size utilized in the rat experiment and presented notable discrepancies in SBP and DBP when comparing the two negative controls (saline solution and water). The flame jellyfish (*R. esculentum*) was hydrolyzed with pepsin and papain and displayed ACE inhibitory activity in vitro; the hydrolysate significantly reduced SBP in SHRs (200, 400, and 800 mg/kg) as a single dose and over a five week feeding period [74]. Another study evaluated the anti-hypertensive effect of long-term oral administration of collagen peptides from the flame jellyfish [50]. However, this study was performed in Wistar strain rats, a breed normally used as a normotensive control. 

Subsequently to an in vitro study of *S. clava* [46], the isolated pentapeptide was orally administered (100 mg/kg bw) to SHRs and this resulted in a significant reduction of SBP compared to the control group given saline solution [75]. However, the sample groups were not disclosed in the article. A more recent study investigated the hypertensive effect of a known antioxidant peptide and reported that the activity remained unchanged after treatment with gastrointestinal enzymes [76]. A single-dose treatment (40 mg/kg bw) of the peptide in SHRS led to a significant reduction in SPB, similar to captopril, compared to a negative control with saline solution. The study further revealed that the peptide in question demonstrated a robust resistance to degradation by gastrointestinal enzymes. Additionally, in silico simulations showed that the peptide enhanced the stabilization of the ACE complex through its interaction with the amino acids’ leucine and tryptophan.

### 1.5. Clinical Trials

The current body of clinical trials investigating the anti-hypertensive effects of peptides and proteins sourced from the marine invertebrate phyla selected for our study is limited and requires further exploration. Moreover, comprehensive data on the safety of these peptides and proteins, including aspects such as toxicity, allergenicity, gastrointestinal stability, and bioavailability is absent, and only a single study met the inclusion criteria of this review (Table 3). In a randomized, placebo-controlled, double-blind study, 34 patients with type 2 diabetes mellitus and hypertension, received 500 mg/day of hydrolysates from the ascidian tunicate *S. clava* over four weeks [77]. After four weeks, the SBP and DBP of the patients had decreased significantly compared to the placebo group (which was undisclosed). The authors reported no adverse effects, including any biochemical and hematological parameters. However, as the authors note, the use of food-grade enzymes in producing the hydrolysate suggests it may be relatively safe, but there is still a necessity to explore potential adverse effects associated with long-term use in a broader population of patients with hypertension.

## 2. Materials and Methods

**Figure 1:**
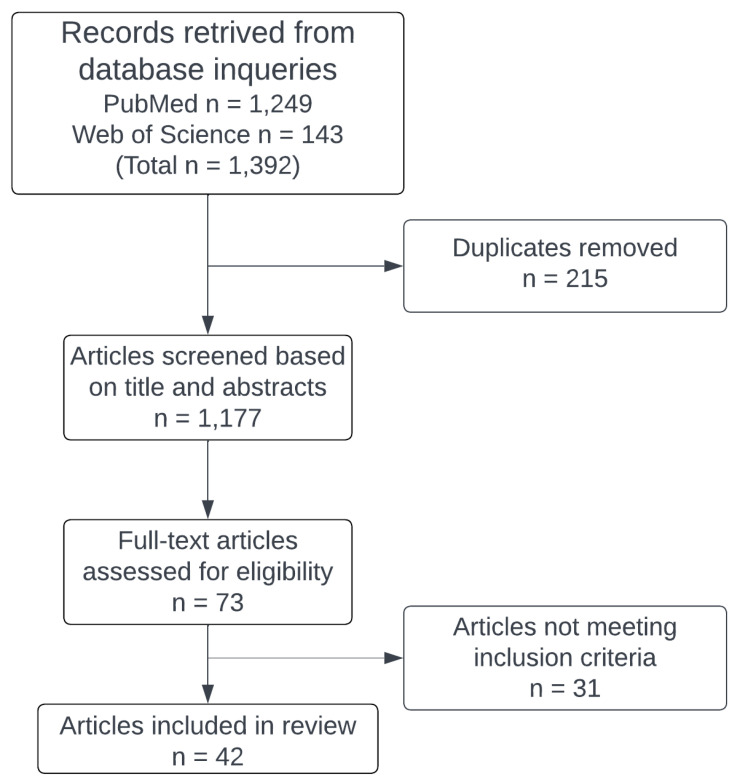
Overview of the selection process of articles included in this review. Flow chart created in Lucid (lucid.co, https://community.lucid.co/admin-questions-2/question-publishing-lucidchart-figures-in-academic-journals-and-citation-5969, accessed on 1 February 2024).

A systematic literature search was performed within the PubMed and Web of Science databases to review literature on selected marine invertebrate phyla. Specific keywords, such as “Mollusca”, “Mollusks”, “Echinodermata”, “Echinoderms”, “Porifera”, “Sponges”, “Cnidaria”, “Cnidarians”, “Chordata”, “Urochordata”, and “Tunicates”, were used in combination (AND) with “Hypertension”, “Hypertensive”, “Antihypertension”, and “Anti-hypertensive”. Rayyan, a web and mobile app for systematic reviews [78], was utilized for the selection process. Inclusion criteria required articles in English, accessible, and indexed in the aforementioned databases, part of the marine phyla of interest, and focusing on hypertension related to proteins or peptides. Articles not meeting these criteria were excluded. Out of 1177 unique articles identified as of June 2023, 42 met the inclusion criteria for this review (Figure 1).

## 3. Conclusions

The increased harvesting of low-trophic marine organisms has made it interesting and relevant to map anti-hypertensive peptides and proteins from these resources for use in functional foods, dietary supplements, or drug synthesis. The ongoing prevalence of adverse reactions associated with currently utilized anti-hypertensive medications has significantly elevated the importance of researching and developing novel anti-hypertensive peptides. Most of the research conducted in this field is predominantly biochemical, with a lesser emphasis on cell-based assays, animal models, and clinical trials. Biochemical in vitro assays for ACE inhibitory activity are commonly used to investigate anti-hypertensive potential, since these assays are affordable, timesaving, and readily accessible. Nonetheless, a diverse array of methods and models used to investigate ACE activity impedes the direct comparison of inhibitory activity across various studies. This lack of standardization presents a challenge in the field, underlining the need for a more unified approach. In vitro cell models enable control over experimental conditions and the exploration of mechanisms and molecular interactions. In silico studies are more economical and environmentally friendly; however, they demand specific expertise and resources, and, without a subsequent peptide synthesis, this approach may overlook dynamic factors inherent in conventional screening methods. While several studies have utilized rats selectively bred for hypertension to examine the anti-hypertensive effects of peptides and proteins, research involving human clinical trials on anti-hypertensive peptides and proteins derived from the specified marine invertebrate phyla remain limited. Before any new anti-hypertensive drug derived from marine invertebrates can be introduced to the market, addressing the lack of extensive data concerning its safety profile, including toxicity and allergenic potential, is imperative. Furthermore, critical considerations, like stability and bioavailability, must be thoroughly investigated and resolved. This review compiles current studies and emphasizes the potential of anti-hypertensive proteins or peptides derived from marine invertebrates, a field that has yet to receive sufficient attention to date. Notably, the Porifera phylum is scarcely represented, with only a single publication in the biochemical section and none in the subsequent sections. While the bulk of the research we reviewed focuses on edible mollusks and their by-products, the extensive variety of species within this understudied phylum underscores the vast potential for further global research. The insights presented herein could lay a robust foundation for expansive research into these valuable marine species. However, it is crucial to balance the exploration of these resources with sustainable practices. Increased reliance on in silico studies and the exploration of synthetic functional peptides could mitigate the risk of overharvesting marine resources in the pursuit of new anti-hypertensive compounds.

## Figures and Tables

**Table 1:** This table summarizes the biochemical studies investigating the anti-hypertensive effect from hydrolysates or peptides derived from selected phyla.

Common Name	Scientific Name	Tissue	Method	Control	IC_50_-Values or % ACE Inhibition	Peptide Sequence	Reference
**Mollusca**							
Mediterranean mussel	*Mytilus galloprovincialis*	Meat	EH with subtilisin and corolase	-	3.7 ± 0.22 and 1.0 ± 0.56 mg/mL	-	[27]
Blue mussel	*Mytilus edulis*	Meat from co-products *	EH with Alcalase^®^, Alcalase^®^ + flavourzyme, corolase PP or Promod 144 MG	Unhydrolyzed protein	1.13–3.34 mg/mL	-	[28]
Blue mussel	*Mytilus edulis*	Byssus	EH with Alcalase^®^, Alcalase^®^ + flavourzyme, corolase PP, Promod 144 MG or *An*-PEP	Unhydrolyzed protein	0.77–1.37 mg/mL	-	[29]
Akoya Pearl oyster	*Pinctada fucata*	Shell	EH with orientase 22 BF	-	5.82 ± 0.56 μg/mL	GVGSPY	[30]
Akoya pearl oyster	*Pinctada fucata*	Meat	EH with Alcalase^®^	*-*	18.34 and 116.26 μM	FRVW and LPYY	[31]
Chinese Venus	*Cyclina sinensis*	Meat	EH with trypsin	-	0.789 mM	WPMGF	[32]
West African mud creeper and Nigerian periwinkles	*Tympanotonus fuscatus* var. *radula* and *Pachymelania aurita*	Meat and hemolymph	Simulated GI digestion model with pepsin, trypsin, and chymotrypsin	Captopril	54.93 ± 2.83, 291.7 ± 8.6, 65.2 ± 6.4, and 301.9 ± 59.1 μg/mL	-	[33]
Scallop	*Chlamys Farreri*	Mantle	EH with neutral protease and trypsin	-	10.28 mg/mL	-	[34]
Comb Pen Shell	*Atrina pectinate*	Edible parts	SCWH	Captopril 1%	85.85 ± 0.67, 84.55 ± 0.18, and 82.15 ± 0.85%	-	[35]
Comb Pen Shell	*Atrina pectinate*	Viscera	SCWH	Captopril 0.1%	96.77 ± 0.14–92.16 ± 0.04%	-	[36]
**Porifera**							
Sponge	*Stylotella aurantium*	Whole body	EH with pepsin	-	273.2 and 306.4 μM	YR and IR	[37]
**Echinodermata**							
Stonefish	*Actinopyga lecanora*	Gutted whole body	EH with Alcalase^®^, bromelain, trypsin, papain, pepsin or flavourzyme	Captopril	1.50, 1.73, 2.04, 2.18, 2.31, and 2.54 mg/mL	-	[38]
Indonesian sea cucumbers	*Holothuria atra, Holothuria leucospilota, and Bohadschia marmorata*	Gutted whole body	EH with Alcalase^®^ or bromelain	-	0.32–0.58 mg/mL and 0.64–0.79 mg/mL	-	[39]
Sea cucumber	*Argyrosomus japonicus*	Whole body	EH with Alcalase^®^	-	58.87–80.38%	-	[40]
Sea cucumber	*Acaudina molpadioidea*	Body wall	EH with trypsin, and papain	Captopril	8.18 and 13.16 μM	PNVA and PNLG	[41]
**Cnidaria**							
Box Jellyfish	*Chiropsalmus quadrigatus*	Venom	EH with pepsin and papain	-	2.03 μM	ACPGPNPGRP	[42]
Flame Jellyfish	*Rhopilema esculentum*	Whole body	Compound proteinase AQ hydrolysis	EH with other enzymes	8.4, 23.42, 21.15, and 19.11 μmol/L	VGPY, FTYVPG, FTYVPGA, and FQAVWAG	[43]
Flame Jellyfish	*Rhopilema esculentum*	Collagen	EH with Alcalase^®^	-	43 μg/mL	-	[44]
**Chordata**							
Solitary tunicate	*Styela plicata*	Tissue	EH with Protamex	EH with other enzymes	24.7 μM	MLLCS	[45]
Club tunicate	*Styela clava*	Flesh tissue	EH with Protamex	EH with other enzymes	37.1 μM	AHIII	[46]

EH = enzymatic hydrolysis, GI = gastro-intestinal, SCWH = subcritical water hydrolysis, * Co-products include byssus, shell, and meat from undersized, cracked, and fouled mussels.

**Table 2:** This table summarizes cellular and in silico studies investigating the anti-hypertensive effect of selected phyla.

Common Name	Scientific Name	Tissue	Method	Control or Databased Used	IC_50_-Values or % ACE Inhibition	Peptide Sequence	Reference
**Mollusca**							
Blue mussel	*Mytilus edulis*	Proteins	Cell model HUVECs	Captopril and norepinephrine	0.77 ± 0.020, 0.19 ± 0.010, and 0.32 ± 0.017 mg/mL	IK, YEGDP, and SWISS	[53]
Deep sea snail	*Volutharpa ampullacea perryi*	Edible parts	Cell model HUVECs	Bradykinin enhancer B, octapeptide angiotensin II, and lisinopril	76.34 ± 0.79 and approximately 40%	IVTNWDDMEK and VGPAGPRG	[54]
Jumbo squid	*Dosidicus giga*	Skin	In silico with pepsin and trypsin	PeptideRanker	-	-	[55]
Japanese flying squid	*Todarodes pacificus*	Myosin heavy chain	In silico and in vitro with papain, ficin, and in combination	BIOPEP-UWM and AHTpin	pIC_50_ = 4.58 and 4.41	IIY and NPPK	[56]
Pacific oyster	*Crassostrea gigas*	Large proteins	In silico with pepsin, trypsin, and chemo-trypsin	AHTpDB	-	-	[57]

**Table 3:** This table summarizes pre-clinical(animal) and clinical studies that have investigated the anti-hypertensive effect of hydrolysates or peptides obtained from selected phyla.

Common Name	Scientific Name	Tissue	Method	Control	IC_50_-Values or ACE Inhibition %	Peptide Sequence	Animal Model	Dosage	Duration	Reference
**Mollusca**										
Blue mussel	*Mytilus edulis*	Muscle	Fermentation 6 months	Captopril and saline solution	19.34 μg/mL	EVMAGNLYPG	SHR	10 mg/kg bw, oral injection *	9 h	[62]
Blue mussel	*Mytilus edulis*	Muscle	EH with Alcalase^®^	Water		VW, LGW, and MVWT	SHR	10 or 20 mg/kg/day hydrolysate, daily oral injection	28 days	[63]
Abalone	*Haliotidae rubra*	Viscera	Fermentation with *Lactiplantibacillus pentosus* SN001	Standard diet	80%	-	SHRs	5% hydrolysate in the diet, ad libitum	9 weeks	[64]
Japanese littleneck clam	*Ruditapes phillippinarum*	Meat	Fermentation with *Bacillus natto*	Undisclosed model group	8.16 μM	VISDEDGVTH	SD rats	8 mg/ kg bw and 32 mg/kg bw peptide, oral gavage *	6 days	[65]
Japanese littleneck clam	*Ruditapes phillippinarum*	Meat	Fermentation with *Bacillus natto*	10 mg/ kg bw saline and captopril	-	-	SHRs	100 mg/ kg bw peptide, daily oral gavage	8 weeks	[66]
Scallop	*Chlamys farreri*	Skirt	Fermentation with *Bacillus natto*	1 mL/kg solution and 10 mg/kg captopril	0.12 ± 0.01 mg/mL	AGFAGDDAPR, CDVDIR, IIAPPER, IWHHTFYNGLR and GIQTAVR	SHRs	25, 50, or 100 mg/kg fraction in the diet, ad libitum	24 h and 8 weeks	[67]
Oyster	-	Meat	EH with aloase and pancitase	Control diet without hydrolysate	-	-	SHRs	5% oyster extracts in the diet, ad libitum	4 weeks	[68]
Oyster	*Crassostrea gigas*	Whole body and striate muscle	EH with trypsin	Control diet without hydrolysate	143 and 28 nmol/mL	DLTDY and DY	SHRs	50, 100, and 1000 mg/kg day, single oral injection and in the diet, ad libitum	6 h, 24 h, and 9 weeks	[69]
Oyster	-	Cross-linked protein	EH	Untreated SHRs, Sardine hydrolysate and captopril	16.7, 29.0, 51.5, 68.2, and 93.9 μM	TAY, VK, KY, FYN, and YA	SHRs	Hydrolysate, single oral gavage	24 h	[70]
Pearl oyster	*Pinctada fucata martensii*	Meat	EH with alkaline protease	10 mg/kg captopril and saline solution	458 ± 3.24 and 109 ± 1.45 μM	HLHT and GWA	SD rats	10 mg/kg bw hydrolysate, single intravenous administration	45 min	[71]
**Echinodermata**										
Sea cucumber	*Acaudina molpadioidea*	Body wall protein	EH with bromelain and Alcalase^®^	3 μM/kg captopril and saline solution	15.9 and 4.5 μM	MEGAQEAQGD	SHRs	3 μM/kg, one-shot oral injection	6 h	[72]
Sea cucumber	*Actinopyga lecanora*	Muscle	EH with bromelain	50 mg/kg captopril, water, and saline solution	-	-	SD rats	200, 400, and 800 mg/kg bw, single oral gavage	3 h	[73]
**Cnidaria**										
Flame jellyfish	*Rhopilema esculentum*	Flesh	Two-step EH with pepsin and papain	50 mg/kg captopril and distilled water	1.28 mg/mL	-	SHRs	200, 400, and 800 mg/kg hydrolysate, single oral gavage, and daily oral gavage	8 h and 5-weeks	[74]
Flame jellyfish	*Rhopilema esculentum*	Collagen	EH with Alcalase^®^	Captopril and control diet	43 μg/mL	-	Wistar strain rats	25 and 100 mg/kg bw, daily oral gavage	4 weeks	[50]
**Chordata**										
Club tunicate	*Styela clava*	Flesh	EH with Protamex	30 mg/kg bw amlodipine and saline solution	-	AHIII	SHRs and SD rats	100 mg/kg bw peptide, single oral gavage	24 h	[75]
Club tunicate	*Styela clava*	Flesh	Synthesized	Captopril and saline solution	16.4 ± 0.45 μM	LWHTH	SHRs	40 mg/kg bw peptide, single oral injection	9 h	[76]
Club tunicate	*Styela clava*	Flesh	Randomized placebo-controlled double-blind study	Not disclosed	-	-	Human	500 mg/day, capsule	4 weeks	[77]

bw = body weight, EH = enzymatic hydrolysis, SHR = spontaneously hypertensive rats, SD rats = Sprague Dawley rats. * Periodicity not reported.
